# Genetic Evaluation of Late-Onset Hypertrophic Cardiomyopathy: An Autobiographical Case Report

**DOI:** 10.7759/cureus.23349

**Published:** 2022-03-21

**Authors:** Simon Carlo, Laura F Rodríguez-Fernández, Fabiola A Benítez Ríos, Norma J Arciniegas-Medina, Hector Martínez-González

**Affiliations:** 1 Biochemistry/Pediatrics/Psychiatry, Ponce Health Sciences University, Ponce, PRI; 2 Biochemistry, Ponce Health Sciences University, Ponce, PRI; 3 Pediatrics, Ponce Health Sciences University, Ponce, PRI; 4 Cardiology, Mayaguez Medical Center, Mayaguez, PRI

**Keywords:** late onset, hypertrophic cardiomyopathy, cardiomyopathy, genetics, autobiographical case report

## Abstract

Cardiomyopathy, also known as a pathology with a cardiovascular cause, can be further differentiated into multiple categories including genetic. Strong correlations between genetic mutations in sarcomeric proteins and presentation of cardiomyopathies have been made. This case report describes the clinical diagnosis of my late-onset hypertrophic cardiomyopathy, which was brought upon by symptoms of chest pain and palpitations that started approximately two years ago and had mostly gone unnoticed during this period. As a geneticist, I decided to undergo genetic test upon diagnosis. These tests found a heterozygous variant of uncertain significance (VUS) in the *ALPK3* gene, c.399dup (p.Gly134ArgfsTer30), and a heterozygous c.7552G>A (p.Val2518Ile) VUS in the desmoplakin (*DSP*) gene. This autobiographical case report hopes to shed light on the importance of genetic screening in the search for the etiology of clinical symptoms.

## Introduction

Cardiomyopathies are defined as pathologies that are known to have a cardiovascular cause [1]. They can be differentiated into primary categories, such as genetic, acquired, or mixed, or secondary categories, where involvement of the myocardium results from a systemic condition. These categories may develop into different phenotypes, which result in hypertrophic, dilated, or restrictive cardiomyopathies [1]. These distinct phenotypes can result in manifestations ranging from microscopic alterations in the cardiac myocytes to fulminant cardiac failure [1]. Hypertrophic cardiomyopathy is the most common hereditary disease of the heart with a prevalence of one in every 500 individuals [1,2]. It is defined as left ventricular hypertrophy without chamber dilation and with predominating septal thickening that may lead to left ventricular outflow tract obstruction or mitral valve dysfunction [1]. Common signs and symptoms of this condition are dyspnea, angina pectoris under stress, dizziness, palpitations, occasional syncope, and a systolic murmur that increases in intensity during Valsalva maneuvers or short-acting preload reducers (ex. acute nitrate therapy) [1,2]. Hypertrophic cardiomyopathy typically presents with atypical chest pain and sudden cardiac death or is diagnosed during the family screening of asymptomatic patients [1]. A diagnostic examination can also show left ventricular hypertrophy and Q waves on electrocardiography as well as hypertrophy of the left ventricle coupled with a reduction in the ventricular chamber volume in echocardiography [1]. The main etiology behind this pathology is autosomal dominant mutations in genes that code for sarcomeric proteins such as beta-myosin heavy chain, cardiac myosin binding protein C, cardiac troponin T, or genes that are involved in the differentiation of cardiac myocytes such as *ALPK3 *[1-3]. These autosomal dominant mutations can be of variable penetrance [2]. In terms of arrhythmogenic right ventricular cardiomyopathy (ARVC), it is classified as an inherited genetic defect that has a prevalence of one in 1,000-5,000 individuals [1]. ARVC involves the desmosomal proteins whose main role is cell adhesion [4]. This defect results in a fibrofatty infiltration and replacement of the myocardium mainly in the right ventricle which leads to the thinning and aneurysmal dilatation of the ventricular muscle wall [1,4]. Histologically, this can be observed to involve an immunologically mediated process due to the inflammatory infiltrates, composed predominantly of T lymphocytes, identified with dying myocytes [4]. In terms of etiologies, several gene mutations, including different proteins such as desmoplakin (*DSP*), desmoglein 2 (*DSG2*), desmocollin 2 (*DSC2*), and plakophilin 2 (*PKP2*) [4], have been identified to be involved in this disease. The clinical manifestations of ARVC usually become most noticeable between the second and fourth decades of life; the first manifestation sometimes being sudden cardiac death [4]. The presentation can range from asymptomatic to ventricular arrhythmias, cardiac arrest, palpitations, and syncopal episodes, the most common being the last two mentioned [4,5]. The clinical signs and symptoms can include chest pain, transient alterations in the ST segment and inversion of the T waves, elevated levels of muscle enzymes, and may or may not have ventricular arrhythmias with a left bundle branch block pattern [4,5]. In addition, abnormalities in the right ventricle can be observed in imaging studies. These include global dilatation, dysfunction, and motion abnormalities of the right ventricle; the septum and left ventricle, if involved, are affected to a lesser extent [4]. In this case report, I will be discussing two of these genes previously mentioned: *ALPK3* and *DSP*. Minimal data on the *ALPK3* gene has led to the belief that it is involved in the phosphorylation of cardiac relevant transcription factors and cardiomyocyte differentiation, with possible abnormal calcium handling by these mutated myocytes [3]. The *DSP* gene codes for desmoplakin, a protein that has been demonstrated to be a part of desmosomes that form the intercellular junctions [6].

## Case presentation

**Figure 1:**
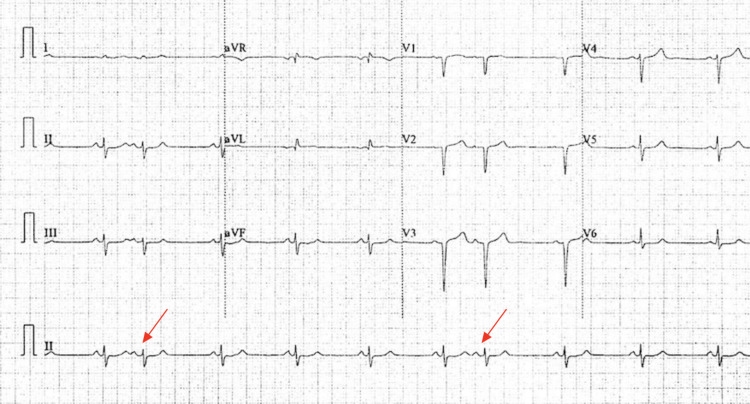
EKG showing sinus bradycardia of 58 bpm with sinus arrhythmia. Arrows indicate premature atrial contractions EKG, electrocardiogram.

I, a 60-year-old Hispanic male, started experiencing chest pain (similar to angina pectoris) and palpitations at the age of 58. Around September 2021, at the age of 59, I experienced a nocturnal stabbing chest pain that lasted for 45 minutes and was not accompanied by diaphoresis or pallor associated with myocardial infarctions. Following this episode, I consulted a cardiologist who suggested for multiple preliminary tests such as an echocardiogram, electrocardiogram (EKG), a Holter test, and an exercise stress test. The findings of the echocardiogram were notable for moderate-concentric left ventricular hypertrophy (LVIDs 2.2 cm, LVIDd 3.6 cm, LVPWd 1.5 cm, and LVPWs 1.8 cm) with a normal-sized left atrium and diastolic function. The ejection fraction was determined to be >55% with a normal left ventricular systolic function. Additionally, no regional wall motion abnormalities were noted, and the transmitral spectral Doppler flow pattern was found to be normal for my age. The right atrium and ventricle were normal in size and thickness. The mitral, tricuspid, and aortic valves were normal; the pulmonic valve was not well visualized. No stenosis was identified in any of the valves, but there was trace mitral and tricuspid regurgitation. The EKG identified sinus bradycardia of 58 beats per minute (bpm) with sinus arrhythmia and a left anterior hemiblock (Figure 1). The Holter test revealed a mean heart rate of 65 bpm with an underlying sinus rhythm. Additionally, no arrhythmias, atrioventricular blocks, ventricular tachycardia, or ventricular fibrillation were identified, which concluded that the findings were within normal Holter parameters. The exercise stress test demonstrated upsloping ST changes and an unsustained ventricular tachycardia with few premature atrial contractions. The maximum predicted heart rate (MPHR) achieved was 188 bpm (117% of HER MPHR) with a maximum blood pressure of 140/80 mmHg, and these responses were classified as adequate. In terms of family history, my maternal grandmother and uncle had valvular problems, whereas my paternal grandfather suffered several infarctions. All of these findings led to the diagnosis of hypertrophic cardiomyopathy. As a geneticist, I decided to conduct genetic tests. Sequence analysis and deletion/duplication testing of 168 genes in the Invitae Arrhythmia and Cardiomyopathy Comprehensive Panel with Preliminary-evidence Genes for Arrhythmia and Cardiomyopathy and Sudden Unexpected Death in Epilepsy (SUDEP) Genes add-ons were performed to detect possible genetic etiology for the current diagnosis. Genetic studies identified the heterozygous *ALPK3* variant of uncertain significance (VUS) 15-85360470-G-GC, p.Gly134ArgfsTer30 (NM_020778.4:c.399dup), and the heterozygous *DSP* VUS 6-7585047-G-A, p.Val2518Ile (NM_004415.2:c.7552G>A) [7]. As of now, I experience chest pains, palpitations, and random arrhythmias that are not related to any specific event and may occur during rest. No specific treatment or lifestyle changes were recommended as I regularly exercise daily, have a balanced diet, and have a normal BMI of 22.3 kg/m^2^ (W: 138 lbs, H: 66 in) since the onset of symptoms. The only recommendation was follow-up visits with the cardiologist every six months or when severe symptoms occur. Possible functional genetic studies are under consideration to conclude a direct correlation between symptoms and the mutations found.

## Discussion

As described above, I report my case of a 60-year-old Hispanic male portraying a late-onset, potentially genetic cardiomyopathy. The determination of the disease as late-onset is made by my lifelong asymptomatic presentation up until the age of 58. Genetic testing concluded the presence of two VUSs: one on the *ALPK3* gene and another in the* DSP* gene. The *ALPK3* gene, which codes for alpha kinase 3, is located at chromosome 15q25.3 and consists of 14 exons [8]. The main function of this gene is to enable ATP binding and enabling protein serine kinase, serine/threonine kinase, and serine/threonine/tyrosine kinase activity [9]. Therefore, it is involved in the development of cardiac muscle, the heart, and protein phosphorylation [9]. As previously mentioned, it was found that I have an* ALPK3* 15-85360470-G-GC, p.Gly134ArgfsTer30 (NM_020778.4:c.399dup), a monoallelic heterozygous variant that creates a premature translational stop signal that is expected to result in an absent or disrupted protein product. Previously reported data demonstrate that alpha kinase 3-deficient cardiomyocytes tend to have abnormal calcium handling due to disorganized sarcomeres and intercalated discs [10]. This strongly suggests an explanation for the association of the *ALPK3* gene with autosomal-recessive dilated cardiomyopathy and autosomal-dominant and -recessive hypertrophic cardiomyopathy. It also puts forward the consideration of a strong connection between the mutation and my current phenotype. The other mutation that was identified was the* DSP* VUS 6-7585047-G-A, p.Val2518Ile (NM_004415.2:c.7552G>A). The *DSP *gene is located on chromosome 6p24.3, consists of 24 exons [11,12], and encodes for the protein desmoplakin. This protein consists of 2872 amino acids and is part of the intercellular junction known as desmosomes [6]. Desmosomes form sites of anchorage of the membrane for intermediate filaments in cells; they also participate in an interaction with the plasma membrane of the adjacent cells. These interactions provide intercellular adhesions and impart resilience and tensile strength to the epithelium [12]. Several mutations have been identified in this gene, which results in different phenotypes. The possible phenotypes include: ARVC; dilated cardiomyopathy (DCM) with woolly hair and keratoderma; DCM with woolly hair, keratoderma, and tooth agenesis; epidermolysis bullosa, lethal acantholytic; keratosis palmoplantaris striata II; skin fragility-woolly hair syndrome; and *DSP* cardiomyopathy [6,13]. Recent investigations performed by Smith et al. stated that there is a specific type of *DSP* cardiomyopathy related to truncating or missense mutations in this specific gene [13]. They concluded that the *DSP* cardiomyopathy is a different type of arrhythmogenic cardiomyopathy that is characterized by episodic injury to the myocardium that leads to progressive fibrosis of the left ventricle and systolic dysfunction with an ejection fraction of less than 55% [13]. According to the Invitae report, genetic testing companies have developed algorithms to foresee the possible implications of the valine for isoleucine substitution mutation present in the c.7552G>A (p.Val2518Ile) variant. In terms of the structure and function of the protein, these algorithms suggest that this variant would likely be tolerated due to the nature of both amino acids being neutral and nonpolar. Since the fact that the amount of evidence that has been reported on this specific variant of the *DSP* gene is insufficient, it has been classified as a VUS.

## Conclusions

This case report highlights the importance of genetic testing when non-specific cardiac-related symptoms appear at a later age. Genetic assessment of older patients is not common and should be encouraged in some challenging cases where a clear etiology is not easily identified. The implications of genetic evaluation in older populations are noteworthy, as they could lead to better identification of clinical knowledge regarding many diseases. In addition, my report suggests an association between monoallelic *ALPK3* and *DSP* gene variant carriers and the presentation of cardiomyopathic phenotypes even when manifestations present at a later age and are not as severe as they have previously been reported in the literature. The possibility of functional studies to conclude direct correlations between a patient’s genotypic and phenotypic profiles is also an important fact to address in the future.
